# Quantification of the rat spinal microglial response to peripheral nerve injury as revealed by immunohistochemical image analysis and flow cytometry

**DOI:** 10.1016/j.jneumeth.2007.04.013

**Published:** 2007-08-30

**Authors:** J. Blackbeard, K.P. O’Dea, V.C.J. Wallace, A. Segerdahl, T. Pheby, M. Takata, M.J. Field, A.S.C. Rice

**Affiliations:** a Department of Anaesthetics, Pain Medicine and Intensive Care, Faculty of Medicine, Imperial College London, Chelsea and Westminster Hospital Campus, 369 Fulham Road, London SW10 9NH, UK; b Translational Medicine Pain Therapeutics, Pfizer Global Research & Development, Sandwich, UK

**Keywords:** Microglia, Nerve injury, Spinal cord, OX42, ED9, Immunohistochemistry, Flow cytometry, Image analysis

## Abstract

Microgliosis is implicated in the pathophysiology of several neurological disorders, including neuropathic pain. Consequently, perturbation of microgliosis is a mechanistic and drug development target in neuropathic pain, which highlights the requirement for specific, sensitive and reproducible methods of microgliosis measurement. In this study, we used the spinal microgliosis associated with L5 spinal nerve transection and minocycline-induced attenuation thereof to: (1) evaluate novel software based semi-quantitative image analysis paradigms for the assessment of immunohistochemical images. Microgliosis was revealed by immunoreactivity to OX42. Several image analysis paradigms were assessed and compared to a previously validated subjective categorical rating scale. This comparison revealed that grey scale measurement of the proportion of a defined area of spinal cord occupied by OX42 immunoreactive cells is a robust image analysis paradigm. (2) Develop and validate a flow cytometric approach for quantification of spinal microgliosis. The flow cytometric technique reliably quantified microgliosis in spinal cord cell suspensions, using OX42 and ED9 immunoreactivity to identify microglia.

The results suggest that image analysis of immunohistochemical revelation of microgliosis reliably detects the spinal microgliosis in response to peripheral nerve injury and pharmacological attenuation thereof. In addition, flow cytometry provides an alternative approach for quantitative analysis of spinal microgliosis elicited by nerve injury.

## 1 Introduction

Microglia constitute 5–10% of the glial population of the adult central nervous system (CNS) and are considered to be derived from a macrophage lineage (for review see Gehrmann and Kreutzberg, 1994). There is an evolving body of evidence implicating microglia in the pathophysiology of a spectrum of neurological disorders, including neuropathic pain. Under physiological conditions, spinal cord microglia are in a ‘resting’ state and do not actively influence nociceptive processing (Kreutzberg, 1996; Watkins et al., 2001b; Watkins and Maier, 2002, 2003; Tsuda et al., 2005). However, there is increasing evidence that following peripheral nerve lesions, spinal microglia migrate to the relevant spinal segments, become activated and subsequently interact with sensory neurones in a manner which appears to be fundamental to the generation of neuropathic pain (Watkins et al., 2001a, 2001b; Coull et al., 2003, 2005; Tsuda et al., 2005). Several characteristics of activated microglia have been identified including: morphological changes during which microglia become hypertrophic and adopt a somewhat amoeboid, as opposed to dendritic shape (Kreutzberg, 1996); immunophenotypical changes in which activated microglia exhibit increased expression of several cell-surface markers and receptors that participate in the immune response (Kreutzberg, 1996); proliferation (Gehrmann et al., 1995) and migration to the site of injury (Tsuda et al., 2003), where they release various chemical mediators, including proinflammatory cytokines, or support neuronal recovery through secretion of growth factors and the isolation/removal of neuronal and myelin debris (Kreutzberg, 1996; Bruce-Keller, 1999; Tsuda et al., 2005).

Reliable and sensitive methods for quantification of the size of microglial population numbers and the activation status of those cells are essential not only to ascertain the extent of microglial involvement in pathological processes, such as the spinal response to peripheral nerve disease, but perhaps more critically, for studying the effect of novel therapeutics directed at modulation of the microglial response. Immunohistochemical markers are useful tools in the experimental imaging of microglia. Antibodies commonly used for detecting microglia are: OX42, which recognises the β2 integrins, CD11b and CD11c, the isolectin binding molecule IB4, the microglia specific calcium binding protein IBA-1 and major histocompatibility complex (MHC) class II. All are well-established markers of microglia, the increased expression of which is widely believed to be associated with microglial activation (for review (Streit et al., 1988; Kreutzberg, 1996; Guillemin and Brew, 2004)). *In vitro* cultured rat microglia, but not astrcytes or oligodendrocytes, have also been found to express the signal-regulatory protein (SIRP) CD172a, which is recognised by the ED9 antibody (Adams et al., 1998). However, in addition to expression on cells of myeloid origin, CD172a is also found expressed on the surface of neuronal cells, complicating immunohistochemical analysis of microglia using the ED9 antibody (Adams et al., 1998). In the literature, the most frequently described marker/antibody for detection of microglia and microgliosis is OX42 since the constitutively expressed CD11b is upregulated in parallel to the activation-induced morphological transformation of microglia (Ladeby et al., 2005). However, image analysis methods used to specifically assess OX42-immunoreactivity (IR) as an indicator of microgliosis vary.

Previously described image analysis methods include qualitative observer-based subjective immunohistochemical estimations (Colburn et al., 1997) and/or several variations of semi-quantitative estimations based upon either measurement of pixel intensity, counting of immunopositive cells or more complex stereological methods of quantitatively measuring OX42 immunoreactive cells (Eriksson et al., 1993; Stuesse et al., 2000; Kullberg et al., 2001; Soltys et al., 2001, 2005; Tsuda et al., 2003; Clark et al., 2007). However, the specificity, sensitivity and reproducibility of such methods have not been compared directly and therefore there is little consensuses as to which are the optimal methods for assessment of microgliosis, in the anatomical context of the spinal cord.

The aim of the present study was two-fold. First, we evaluated the utility of various image analysis tools for quantification of spinal microgliosis, revealed by immunohistochemistry (using both enzyme and fluorescent based techniques for visualisation) in naïve, sham operated and L5 spinal nerve transected (SNT) rats. In addition tissue from L5 SNT rats treated with minocycline, an established inhibitor of microglial activation, was examined (Yrjanheikki et al., 1998; Tikka and Koistinaho, 2001). We assessed the specificity (i.e. detection of a true negative result) and the sensitivity (i.e. detection of a true positive result) of each method by comparison to a reference method—a previously published observer-based qualitative categorical rating scale of spinal microgliosis (Colburn et al., 1997). Second, we aimed to identify a flow cytometry based methodology which could be applied to the analysis of nerve injury induced spinal microgliosis. Whereas flow cytometry has been applied extensively and proven to be a valuable tool within the field of neuroimmunolgy, its use in the study of spinal microgliosis within the field of neuroscience, and particularly within the field if pain, has been extremely limited. Previous flow cytometry based investigations of spinal cord microglia have focused predominantly on phenotypical and functional characterisation of this microglial cell population (McCombe et al., 1992; Ford et al., 1995; Sedgwick et al., 1998). However, these studies relied on an initial enrichment step which may not be appropriate for analysis of total microglial. For determining total microglia numbers as well as phenotype within specific regions of the spinal cord, we sought to develop a protocol in which microgliosis could be assessed by flow cytometry with minimal *ex vivo* manipulation of cells. Indirect comparisons between the immunohistochemical and flow cytometric approaches were made in order to determine the optimal method for assessing microgliosis in various experimental conditions.

## 2 Methods

All *in vivo* experiments were approved by the United Kingdom Home Office and performed using 200–250 g male Wistar rats (B&K, UK). Animals were housed, maximum four animals per cage at constant temperature under a 14:10 h light/dark cycle, with free access to food and water.

### 2.1 Surgical procedures and pharmacological intervention

A left L5 spinal nerve transection (SNT), a modification of that described by Kim and Chung (1992), was performed on male Wistar rats (Bridges et al., 2001). Surgery was performed under brief isoflurane anaesthesia (Abbott, UK) and aseptic surgical conditions. Briefly, the left paraspinal muscles were exposed, the L6 transverse process was removed by hemi-laminectomy. The L5 spinal nerve was exposed and identified, lifted and ligated with 4.0 Mersilk suture (Ethicon, Edinburgh, UK) before being transected distal to the spinal cord. Sham surgeries were performed by exposing the L5 spinal nerve as described above, carefully avoiding any damage to the nerve itself. Five L5 SNT animals were treated pre-emptively with 40 mg/kg minocycline (Sigma–Aldrich Co. Ltd., UK) dissolved in sterile saline, administered i.p. once daily commencing 1 h prior to L5 SNT until the animal was sacrificed. All animals undergoing surgical intervention received postoperative analgesia consisting of a single s.c. injection of 0.05 ml 0.5% bupivacaine (Antigen Pharmaceuticals, Dublin, Ireland) to the wound site followed by a one off i.p. administration of carprofen (20%, 0.1 ml/200 g body weight, Pfizer, Sandwich, UK) 2 h later. We could locate no evidence in the literature which would indicate that the, ethically unavoidable, provision of such short term postoperative analgesia would interfere with microgliosis at a time point several days later—to test this would probably be ethically unacceptable certain in the United Kingdom. Certainly, we observe a similar pattern of spinal microgliosis to that reported elsewhere following peripheral nerve injury. At postoperative (PO) day 7 the hind-limb withdrawal response to a punctate mechanical stimulus (Electronic von Frey device, Somedic, Sweden) were performed to assess successful induction of neuropathy-associated hypersensitivity, and in the case of minocycline-treated animals, the attenuation thereof.

### 2.2 Tissue preparation

All animals were perfused-fixed at PO day 7. Under terminal anaesthesia with pentobarbitone, rats were transcardially perfused with 100 ml of cold heparinised saline followed by 300 ml of 4% paraformaldehyde (Sigma) in 0.1 M phosphate buffer, pH 7.4. Following perfusion, lumbar spinal cord segments approximately 100 mm in length were harvested by laminectomy and postfixed in 4% paraformaldehyde fixative for 2 h at 4 °C before being cryoprotected in sucrose in 0.1 M phosphate buffer. Segments of spinal cords were freeze-mounted in OCT embedding medium (VWR, Poole, UK) before 15 μm thick tissue sections from L5 spinal cord (identified according to anatomical landmarks (Molander et al., 1984) were cut transversely and thaw mounted on Superfrost Plus slides (BDH).

### 2.3 Immunohistochemistry

For fluorescence immunohistochemistry, sections were pre-incubated with 0.2% Triton X-100 in PBS, containing 10% normal donkey serum for 1 hour at room temperature. For immunohistochemistry using the avidin-biotin technique, sections were pre-incubated with 0.3% H_2_O_2_ for 30 min. Sections were then incubated overnight with the monoclonal antibody OX42 (low endotoxin, labelling CR3, CD11b/c, donkey anti-mouse labelling microglia; 1:800, Serotec, UK). Visualisation of OX42-IR was achieved using either a fluorescent secondary isothiocyanate (Cy3) (donkey anti-mouse-Cy3 1:600; Jackson ImmunoResearch Laboratories, Westgrove, PA) or an avidin-biotin technique (Vector Labs, UK). To minimise variability in staining, tissue from all treatment groups were run in the same immunohistochemical session. A negative control omitting the primary antibody was performed for all experiments.

### 2.4 Image capture

Images were captured on a Leica DMR microscope, using a green fluorescence filter (rhodamine, 530 nm) or standard light microscopy and captured with a Hamamatsu colour 3CCD video camera. Care was taken to analyse the same regions of the spinal cord dorsal horn as determined by referring to anatomical landmarks (Molander et al., 1984). Image analysis was performed with macros developed using Leica QWin software (QWin Standard V 3.1.0). All fluorescent images were digitally converted into a grey scale image before commencing the analysis. A mean reading of 8–10 spinal cord sections from a single animal representative of each of the four test conditions (naïve, sham, L5 SNT or minocycline-treated L-5 SNT) were analysed and the qualitative and quantitative methods assessed in a blinded fashion. Identical camera and microscope settings were used throughout capturing of images and analysis of the slides. For this study only the spinal cord dorsal horns ipsilateral to injury were compared given the aims of the study and the fact that the nerve injury induced microgliosis will be most pronounced on the ipsilateral side of the spinal cord.

### 2.5 Qualitative analysis

**Fig. 1:**
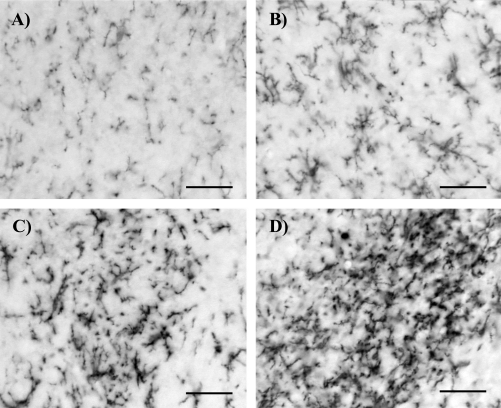
(A–D) Representative images of degrees of microgliosis in sections (15 μm) of L5 spinal dorsal horn as revealed by OX42-IR. (A) Score = ‘0’, resting microglia morphology as observed in naïve spinal cord tissue; (B) score = ‘1’, slight microglial activation typical for L5 SNT sham operated animals; (C) score = ‘2’, intermediate microgliosis characteristic for L5 SNT rats treated pre-emptively with minocycline; (D) score = ‘3’, extensive microgliosis as observed in L5 SNT rats at postoperative day 7 (scale bar 30 μm).

The qualitative rating was derived from a 4 point categorical rating scale developed by Colburn and colleagues (Colburn et al., 1997) which provide an evaluation of microgliosis based on morphological and immunoreactivity changes. The rating criteria are explained in Table 1. This published method was used as the reference for all other methods described below. In order to determine test-retest variability, rating of images was performed on 10 different occasions by the same blinded observer. Representative examples of each score are illustrated in Fig. 1.

### 2.6 Semi-quantitative analysis

#### 2.6.1 Grey scale intensity (GSI)

For each spinal cord section three standardised rectangles (120 μm × 120 μm) were superimposed over the image onto areas of the lateral, central and medial ipsilateral spinal dorsal horn and the mean grey scale intensity (GSI) across each rectangle was measured using two scales: (i) A conventional 255 point grey scale with a two point calibration: 0 (pure black) to 255 (pure white). (ii) An expanded 255 point mid-range grey scale where the value of zero was calibrated against the 50 intensity point and 255 against the 200 intensity point derived from scale i. The rationale behind exploring the utility of the expanded grey scale was to determine whether the excluding values at the extremes of a conventional grey scale would improve the signal and hence improve the sensitivity of this method. We argued that, in biological images, such as those examined here, there will be very few pixels with grey scale values towards the pure white and pure black ends of the conventional scale; most readings would be expected to fall into the mid range and therefore readings taken at the extreme points of the scale would be redundant.

For fluorescence visualisation, the background value, as defined by the grey level intensity in an area of tissue outside the measurement area (i.e. in an area of spinal cord tissue with minimal or no OX42-IR), was subtracted from each intensity measurement and the average of the three readings was calculated. For DAB visualisation the exposure time when capturing the images was set so no significant background staining was present.

#### 2.6.2 Grid method (GM)

As a refinement of the rectangle technique described in Section 2.6.1, the utility of a rectangle with an integral grid was employed in order to account for variation in pixel intensity within the rectangle. Initially the pixel intensity threshold for detection of immunoreactivity was established for a control slide by adjustment until the thin processes of OX42 immunoreactive profiles were detected. Settings were standardised by adjusting the black detection to 75 (fluorescence) and 205 (DAB) and maintained throughout the following analysis. A grid of defined dimensions (420 μm × 260 μm; number of lines *x* = 35 and *y* = 30; line spacing *x* = 12 μm and *y* = 9 μm) was subsequently superimposed onto the image covering the area of the lateral, central and medial dorsal horn ipsilateral to SNT injury. The automated computer software programme then allowed the measurement of several stereological parameters as described in Table 2.

### 2.7 Tissue processing for flow cytometric analysis

All animals were transcardially perfused with 150 ml heparinised PBS under terminal anaesthesia with pentobarbitone (60 mg/kg) at PO day 3, 7, 9 and 14. Following perfusion, the L5 lumbar spinal cord was carefully dissected and separated down the midline. Ipsilateral and contralateral spinal cord segments were processed independently. Each section of harvested tissue was weighed and immediately subjected to mechanical disruption by rapid chopping with a scalpel blade. Enzymatic digestions of disaggregated tissue was performed according to method described by Sedgwick et al. (1991). However, to reduce potential *ex vivo* alterations to surface antigen expression, the digestion step was reduced from 60 to 30 min without any apparent reduction in microglia recovery (data not shown). The minced tissue was enzymatically digested with collagenase type IV (0.5 mg/ml in PBS; Sigma UK) with continual mixing for 30 min at 37 °C. After quenching the digestion by the addition of fluorescence-activated cell sorter (FACS) medium (phosphate-buffered saline, 2% fetal calf serum, 0.1% sodium azide, and 5 mM ethylenediaminetetraacetic acid), the cell suspension was further dissociated through a 40 μm nylon cell strainer (BD Falcon, Oxford, UK) with extensive flushing through of cells with FACS medium. Cell suspensions were centrifuged for 7 min at 400 × *g* and re-suspended to 1 ml in 20% normal goat serum in FACS medium. Throughout the procedure, care was taken to maximise cell recovery.

### 2.8 Flow cytometric analysis of spinal microgliosis

**Fig. 2:**
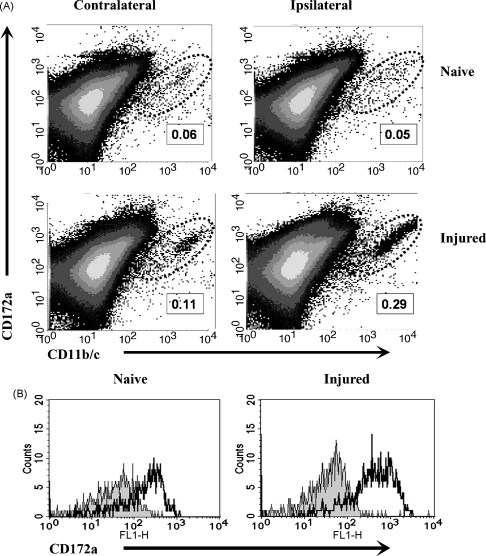
Flow cytometric identification of spinal cord microglia. In the representative experiment shown, ipsilateral and contralateral spinal cord tissue from either naïve or L5 SNT nerve injured rats at postoperative day 7 were processed (see Section 2) for analysis by flow cytometry. (A) Microglia were identified as OX42 (CD11b/c) and ED9 (CD172a) positive events as shown in density plots. Microglia per mg of tissue were then quantified based on numbers of gated OX42/ED9 positive events (see Section 2). The percentages values included on the figure indicate the total percentage of collected cells identified as microglia. (B) Representative fluorescence intensity histograms of ED9 (black line) compared with isotype matched control antibody (grey fill) on OX42 gated ipsilateral spinal cord cells from naïve and injured animals.

Quantification of microglia was based on reactivity with OX42 (CD11b/c) and ED9 (CD172a, SIRP). CD172a is expressed on the surface of neuronal cells and cells of myeloid origin, including *in vitro* cultured microglia (Adams et al., 1998). In view of the necessity to acquire large numbers of total events and therefore the increased potential for false positive events to be present within a single OX42-based gate, we considered that ED9 may be a useful secondary marker to provide additional confirmation of microglia identity. Pilot studies indicated there was co-reactivity between ED9 and the majority of OX42 positive events in spinal cord single cell suspensions (Fig. 2B). The identity of gated cells was further confirmed to be microglial, as opposed to intravascular neutrophils or monocytes, by their lower levels of CD45 expression (Sedgwick et al., 1991) in control and injured rats (data not shown). Cell suspensions (50 μl) were incubated with an equal volume of fluorophore conjugated monoclonal antibodies: APC-conjugated anti-CD11b (OX42), FITC-conjugated anti-CD172a (ED9) and PE-conjugated anti-CD45 (OX1) (Serotec, UK) at a final concentration of 1 in 20 for 30 min at 4 °C in the dark. Cells were washed twice with FACS medium and then analysed with a FACSCalibur flow cytometer and CellQuest Pro software (Beckton and Dickenson, Oxford, UK). A minimum of 5 × 10^5^ events were acquired for each sample. The absolute cell counts in each sample were determined by addition of Perfect-count fluorescent microspheres (Caltag Medsystems, Towcester, UK) prior to sample acquisition and cell numbers calculated according to manufacturer's instructions. Microglial recovery was expressed as cells per mg of excised spinal cord tissue. Additionally, ED9 IR with microglia was quantified on gated OX42 positive cells and expressed as mean fluorescence intensity (MFI).

### 2.9 Statistical methods

For statistical analysis of the quantification methods of microgliosis changes QQ Plot and BOX Plots were used to assess normality of the data (SPSS for Windows version 11.5). To determine statistical significance (*p* < 0.05) of differences between treatment group the Mann–Whitney *U*-test was used. The Pearson correlation was used to correlate the semi-quantitative methods to the qualitative reference method. In order to determine the sensitivity and specificity of each method in detecting and quantifying microgliosis, a dichotomous outcome measure was created using the rule: Naïve rat tissue = no microgliosis. L5 SNT PO 7 rat tissue = maximal microgliosis. Analysis was performed using binary logical regression. The reproducibility of the qualitative reference method was assessed by repeating the rating of the same images 10 times and the results analysed by calculating the intraclass correlation coefficient. Statistical tests for flow cytometric analysis performed using Sigmastat, Jandel Scientific Software (version 2.0) and results are expressed as mean ± S.E.M. For the qualitative reference method and flow cytometry the ipsilateral and contralateral values were compared within each experimental group. Data analysed using ANOVA all pairwise multiple comparison procedures (Student-Newman-Keuls Method). A value of *p* < 0.05 was considered statistically significant.

## 3 Results

### 3.1 Image analysis of immunohistochemically revealed nerve injury induced microgliosis

**Fig. 3:**
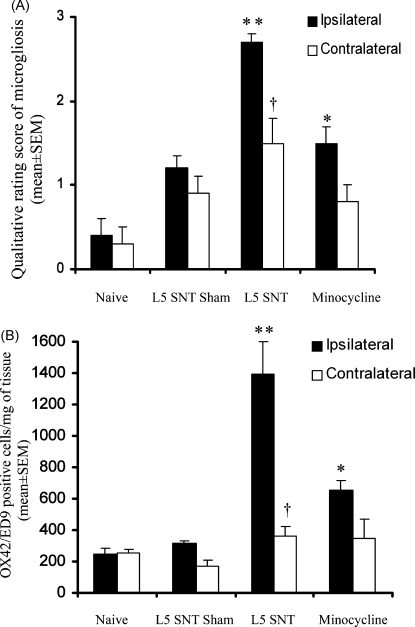
Quantitative analysis of ipsilateral and contralateral microgliosis in L5 lumbar spinal cord tissue from: naïve, sham operated, nerve injured (L5 SNT), and L5 SNT nerve injured animals treated with minocycline (40 mg/kg) at postoperative day 7. (A) Qualitative rating of microgliosis revealed by immunohistochemisty using a 4 point scale raging from 0 (no microgliosis) to 3 (pronounced microgliosis) according to levels of OX42-IR and microglia morphology. Results expressed as mean rating score ± S.E.M. of 8–11 spinal cord sections. (B) Quantitative flow cytometry analysis of total OX42/ED9 positive cells per mg tissue in L5 lumbar spinal cord tissue. Results expressed as mean ± S.E.M. (*n* = 4/group). Differences between naïve and treated groups ipsilateral spinal cord, *p > 0.05, **p > 0.01. Differences between ipsilateral and contralateral spinal cord tissue within each experimental group, ^†^*p* > 0.05.

The qualitative analysis of microgliosis (visualised with the DAB method) was used as the reference method and the degree of microgliosis compared to the semi-quantitative image analysis paradigms (visualised with either DAB or fluorescence). The qualitative rating scale accurately differentiated between the different levels of microgliosis observed in all four experimental conditions (Mann–Whitney *U*-test, *p* < 0.05): As predicted, naïve spinal cord displayed basal levels of OX42-IR and achieved a low qualitative rating score (0.4 ± 0.2) (Fig. 3A). Whereas, spinal cord tissue from L5 SNT, nerve-injured animals displayed significantly higher levels of OX42-IR and achieved a high categorical score (2.7 ± 0.1). Spinal cord from sham operated animals achieved a slightly higher qualitative rating score (1.2 ± 0.2) as compared to naïve, although as expected this was significantly less than that observed in L5 SNT (Fig. 3A). Furthermore, the qualitative rating scale demonstrated that treatment with minocycline was associated with significant reduction in microgliosis in L5 SNT injured animals (categorical score 1.5 ± 0.2 in SNT + minocycline versus 2.7 ± 0.1 in SNT) (Fig. 3A). When the semi-quantification image analysis methods were compared; GSI i + ii and GM i–v (see Table 2 for descriptions) were shown to be sufficiently sensitive to differentiate between the extent of spinal microgliosis observed in L5 SNT and naïve tissue when OX42-IR was revealed using fluorescent secondary antibodies (Mann–Whiney *U*-test, *p* < 0.05). However, with fluorescent visualisation, only method GM i correlated with the results obtained using the categorical scale reference method (*r* = 0.844; *p* < 0.05). In contrast, DAB visualisation of immunoreactivity increased the correlation with the reference method in all analysis paradigms apart from the conventional measure of grey scale intensity (GSI i) and count (GM iv). All correlation values are displayed in Table 3.

### 3.2 Determination of specificity and sensitivity of image analysis methods for immunohistochemically revealed microgliosis

To determine the specificity and sensitivity of the image analysis methods, a dichotomous outcome measure was applied. The results of these tests are shown in Table 4. All tested methods had sensitivity values above 75% and specificity values above 63%. Using DAB as the visualisation technique increased the specificity and sensitivity in all tested methods (except GM i in which sensitivity and specificity was 100% with fluorescence). It should be noted that the expanded 255 point mid-range grey scale, method GSI ii, increased the specificity when used in combination with fluorescence visualisation (Table 4).

### 3.3 Reproducibility of the immunohistochemical methods

The semi-quantitative immunohistochemical analysis methods (GSI i + ii and GM i–v) for detecting the amount of microglia present were fully reproducible due to the analysis relying on a standardised software based paradigm. The reproducibility of the categorical scale, as tested by rating the same images on 10 different occasions, was 80% using DAB visualisation and 64% using fluorescence visualisation of OX42-IR.

### 3.4 Flow cytometric analysis of nerve injury induced microgliosis

**Fig. 4:**
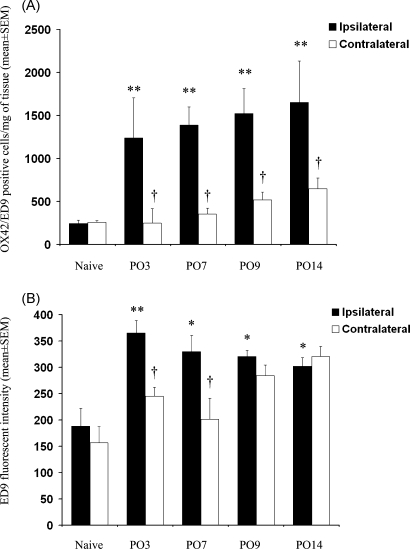
Flow cytometric time course analysis of total OX42/ED9 positive events per mg tissue (A) and ED9 mean fluorescent intensity (MFI) on OX42 positive events (B) in L5 lumbar spinal cord tissue comparing naïve with L5 SNT operated animals at day 3, 7, 9 and 14 postsurgery. Results expressed as mean ± S.E.M. (*n* = 4/group). Differences between naïve and treated group's ipsilateral spinal cord, *p > 0.05, **p > 0.01. Differences between ipsilateral and contralateral spinal cord tissue within each experimental group, ^†^*p* > 0.05.

Absolute numbers of gated cells were determined using fluorescent microsphere beads (Fig. 2A) and numbers of cells per mg of tissue were determined based on the weight of the dissected tissue sections. Ipsilateral spinal cord tissue from L5 SNT nerve-injured rats showed a significant, five-fold increase, in OX42/ED9 gated cells compared with naïve or sham operated rats (230 ± 38 cells/mg tissue for naïve versus 1389 ± 355 cells/mg tissue for L5 SNT animals at PO day 7 (One-Way ANOVA, *p* < 0.01) (Fig. 3B). Pharmacological attenuation of microgliosis was quantifiable in minocycline-treated animals, with a ∼50% reduction in total gated cells in ipsilateral spinal cord tissue (655 ± 58 cells/mg tissue versus 1389 ± 355 cells/mg tissue in L5 SNT alone (One-Way ANOVA *p* < 0.05)) (Fig. 3B). In addition, the ability of flow cytometry to detect time course differences in nerve injury induced microgliosis was assessed in the L5 SNT model (Fig. 4A). These results demonstrate that with this technique microgliosis is detectable at PO day 3, reaching a plateau around PO day 7–9 and remaining elevated until at least PO day 14. These results are consistent with immunohistochemical findings both in terms of time course differences and overall extent of microgliosis (data not shown) (Eriksson et al., 1993; Coyle, 1998). The degree of microgliosis as measured by increased numbers of OX42/ED9 gated events by flow cytometry were also consistent with the immunohistochemical reference method in differentiating between the four groups examined (Fig. 3A).

Using the ratio of ipsilateral versus contralateral degree of microgliosis as an internal control of the method, the flow cytometric results corresponded well with the immunohistochemical reference method with both methods generating ipsilateral/contralateral ratios of ∼1.0 for naïve tissue, ∼1.5 for sham tissue and ∼2.0 for minocycline tissue. The methods did however differ in the magnitude of the ipsilateral/contralateral ratio for L5 SNT tissue in which the immunohistochemical reference method produced a ratio of ∼2 compared to ∼4 for flow cytometry.

In addition to increased numbers of microglia, an overall increase in OX42 and ED9 immunoreactivity was apparent within the total microglia population recovered from nerve injured rats at all investigated time points. To investigate further the potential for using CD172a as a marker for microglia and their activation, mean levels of ED9 immunoreactivity with OX42 positive events were quantified on naïve and L5 SNT derived spinal cords (Fig. 4B). A significant increase in CD172a expression in L5 SNT spinal cord tissue, compared to naïves, occurred as early as PO3 on both ipsilateral and contralateral derived microglia (*p* < 0.05, One-Way ANOVA) (Fig. 4B). However, consistent with regional differences in the magnitude of microglial populations, CD172a expression was significantly higher on ipsilaterally derived microglia (*p* < 0.05, One-Way ANOVA) (Fig. 4B). The differential regional upregulation of CD172a became less evident at PO7 onwards which may be accounted for by reciprocal changes in ipsilateral versus contralateral expression of CD172a over time (Fig. 4B). Detection of CD172a upregulation on contralateral regions, albeit smaller than ipsilateral values at equivalent time points, suggest that CD172a expression could be a more sensitive indicator of (regional and non-regional) early microgliosis using flow cytometric analyses than quantification of the total size of the microglial population.

## 4 Discussion

In this study, we have assessed and cross-validated pre-existing and novel approaches for quantification of spinal cord microgliosis using the L5 SNT model of nerve injury. The immunohistochemical image analysis and flow cytometry methods investigated were based upon the principles of previously reported quantification techniques and were correlated to a standardised qualitative estimation of microglia activation (Colburn et al., 1997). Microglia were identified according to OX42 immunoreactivity given that this has been the antibody of choice for immunohistochemical evaluation of microgliosis (Ladeby et al., 2005) as well as for identification of microglia by flow cytometry. All the immunohistochemical image analysis methods examined were found to differentiate between the microgliosis in nerve injured tissue and the resting microglia status in naïve tissue with a high degree of sensitivity (all values >75%) and specificity (all values >63%). The measure of percentage area covered by microglia in a defined grid (method GM i) demonstrated the greatest specificity and sensitivity when differentiating microgliosis between nerve-injured and naïve spinal cord tissue and was the only image analysis method, which achieved this irrespective of secondary visualisation technique. Additionally, the GM i method displayed the closest correlation with the qualitative rating paradigm. To obtain an absolute quantitative measure of microgliosis we developed a flow cytometry based method for identification and quantification of microglia within dissected spinal cord tissue sections based on reactivity with OX42 and the macrophage marker antibody, ED9. Correlation with the qualitative immunohistochemical image analysis provided validation of the method indicating it represents an alternative approach for investigation of microgliosis during spinal nerve injury.

The role of spinal gliosis in the generation of persistent pain states is becoming increasingly recognised, and represents a divergence from the previously ‘neuro-centric’ view of persistent pain (For review see Wieseler-Frank et al., 2004). Several lines of experimental evidence implicate the activation of spinal cord microglia as a significant component in the pathophysiology of neuropathic pain (Watkins et al., 2001b; Watkins and Maier, 2002; Tsuda et al., 2004; Coull et al., 2005). Although various methods for assessment of microgliosis by immunohistochemistry and image analysis have been described previously in the literature, there is no general consensus regarding the optimal approach for quantification of microgliosis or the most appropriate secondary visualisation technique to apply to each method. The most widely applied immunohistochemical image analysis method is the qualitative rating scale of microglial activation as visualised with biotinylated secondary antibodies (Colburn et al., 1997). We used this qualitative image analysis technique as a reference method since it provides an appropriate estimation of microgliosis, particularly as it incorporates the distinct morphological metamorphosis of microglia from dendritic to amoeboid form as one of the activation criteria. In our hands, this method was found to be: (1) sensitive in discriminating between microgliosis in the four experimental groups examined; (2) capable of detecting pharmacological perturbation and (3) highly reproducible in blinded analysis. Although the qualitative rating scale can provide an accurate measure of microgliosis, the lack of automated image analysis can result in the method being time consuming making it more difficult to incorporate estimation of larger areas of the spinal cord. Moreover, the graded classification system it uses, with a measurement ceiling at a value of 3, is rigid and does not take into account intra-scale differences in activation indices nor provide the degree of precision required for pharmacological attenuation studies. Furthermore, since an observer judgement of the image is an inherent step in the paradigm there is potential for observer bias. One option is to combine the qualitative rating scale using biotinylated visualisation with a semi-quantitative approach, thereby allowing for a comprehensive method of measuring spinal microgliosis by including both a qualitative estimation of morphological changes and semi-quantitative assessment. The benefit of a software based image analysis method is that it involves less subjective evaluation (and thus mitigates against observer derived experimental bias) and is significantly less laborious and time consuming compared to manual observation. Based on the image analysis methodologies evaluated here, we consider that the percentage area of a grid covered by OX42-IR cells (GM i) represents an appropriate and robust image analysis system for assessing microgliosis. Thus, of the image analysis methods tested the GM i method produced the closest correlation with the qualitative rating scale and was found to be the most specific and sensitive when differentiating between microgliosis in the naïve and nerve-injured animal regardless of choice of secondary visualisation. This is a crucial feature since immunohistochemical phenotyping of cellular targets frequently necessitate multiple labelling of the tissue. Consequently, fluorescent labelling is often the primary choice when it comes to image analysis and a method by which image analysis quantification can accurately be execute indispensable. Conversely, measurement of mean grey scale intensity (GSI i + ii) as a quantitative measure of microgliosis was, in our hands, less specific and sensitive than other methods, irrespective of the mode of secondary visualisation. This technique is further limited by not incorporating information relating to changes in microglia cell size. It should be noted that using the biotinylated secondary visualisation technique was found to improve specificity and sensitivity for the majority of immunohistochemical image analysis methods as compared to fluorescence. This may be explained by a low signal-to-noise ratio when using biotinylated visualisation (primarily as a result of lower background) compared to fluorescence. In addition, fluorescent detection will display a reduced linearity that will affect the accuracy of the results. Overall, the results suggest that while fluorescence based techniques are useful for co-localising protein expression within cells or tissues, some caution is required when applying this visualisation technique for qualitative and quantitative image analysis estimations of microgliosis.

Although the immunohistochemical image analysis techniques described here are adequate in giving reliable indications of the degree of microgliosis, they do not permit absolute quantitative measurements of cell density or expression levels of activation markers on individual cells. Flow cytometry based methodologies have been used to study the involvement of local cell-mediated immune responses in various inflammatory diseases of the nervous system in both animal (Ford et al., 1995; Campanella et al., 2002; Wirenfeldt et al., 2005) as well as human studies (Dick et al., 1997). However, despite the quantitative analysis advantages over histological and immunohistochemical approaches, the use of flow cytometry as an analytical tool for measurement/quantification of microgliosis has been limited. We therefore investigated the feasibility of using flow cytometry as a convenient and robust method for analysis of microglia within spinal cord tissue. Previously described flow cytometric analysis of microglia have involved a prior enrichment step using Percoll density gradient separation (Ford et al., 1995; Sedgwick et al., 1998). To obtain a direct quantitative measurement of regional microgliosis, minimise losses to cell yield and potential artefacts related to separation of cells based on their physical properties, we adapted a procedure recently described for quantification of monocytes in lung tissue (O’Dea et al., 2005) omitting the initial enrichment step and performing direct flow cytometric analysis on spinal cord single cell suspensions. Despite the relative paucity of microglia within the total spinal cord cell population, we were able to acquire sufficient numbers of cells to perform quantitative analysis determining absolute numbers of OX42/ED9 stained microglia in spinal cord sections. Importantly, the protocol used successfully characterised the time course of nerve injury induced spinal microgliosis and differentiated between the degrees of microgliosis in all experimental models studied. Furthermore, the protocol presented here was capable of accurately detecting pharmacological perturbation of microgliosis and the data generated correlated overall with the immunohistochemical image analysis reference method.

In addition to an increase in total microglial numbers, an overall increase in OX42 and ED9 IR was also apparent in injured tissue. By quantifying basal and postinjury changes to ED9 binding to OX42 positive cells, in parallel with changes to microglia numbers, we further validated ED9 as a novel tool for flow cytometry based identification of spinal cord microglia and their activation. Detection of CD172a upregulation on contralateral regions, albeit smaller than ipsilateral values at equivalent time points, suggest that CD172a expression may be a sensitive indicator of both regional and non-regional microglia activation status. CD172a (SIRP-a), a member of the signal phosphatase regulatory protein (SIRP) receptor multigene family, is an important regulator of myeloid cell function (Barclay and Brown, 2006). Ligation of CD172a by CD47, which is expressed on a wide range of cells including neurons, inhibits phagocytosis, lipopolysaccharide-induced tumor necrosis factor-alpha release and regulates monocyte and neutrophil migration (Barclay and Brown, 2006). The upregulation of microglia surface CD172a observed in the L5 SNT model could therefore represent a homeostatic response, limiting inflammation and potential injury. This prediction is supported by studies on the CD200-CD200 receptor interaction, a cell-cell communication system closely related to that of CD172a and CD47 (Barclay et al., 2002), which maintains microglia in a quiescent state and has been shown to be instrumental in reducing increases in microglial numbers and activation in mouse models of autoimmune and toxoplasma-induced encephalitis (Hoek et al., 2000; Deckert et al., 2006).

The demonstration of microgliosis by quantification of absolute cell counts in larger dissected tissue, as opposed to the more narrow regional scores on transverse immunohistochemically treated tissue sections used in image analysis indicate that flow cytometry provides not only a complementary, but also a distinct strategy for evaluation of spinal microgliosis. Previous immunohistochemical studies in models of unilateral nerve injury have established a time course for nerve injury related induction of spinal microgliosis as well as a pronounced ipsilateral versus contralateral difference (Eriksson et al., 1993; Coyle, 1998). In agreement with these immunohistochemical findings, the flow cytometric technique described in this study was both able to differentiate time course variations in nerve injury induced microgliosis as well as detecting elevated ipsilateral versus contralateral ratios. Notably, a higher ipsilateral-contralateral ratio in nerve injured rats was observed by flow cytometry (four-fold) as compared to the qualitative analysis method (less than two-fold). This may reflect the different tissue sampling methods, more pronounced differences when limiting analysis to cell numbers or a limitation in detecting differences in microgliosis using the categorical scaling with the ceiling value of 3.

The major advantage of any immunohistochemical technique is the ability to visualise the *in situ* localisation of cells and proteins of interest. Therefore, in the context of microgliosis, immunohistochemistry is particularly useful for determining the anatomical site of microglial activation. By contrast, the described flow cytometry technique provides more precise quantitative information, distinguishing between changes to total microglia numbers and relative changes in levels of protein expression. When used in parallel, flow cytometry and immunohistochemical techniques enable measurement of qualitative, semi-quantitative and absolute quantitative measures of microgliosis and therefore allow a more comprehensive evaluation of the events associated with spinal microgliosis. Such a combined analysis approach (combination of qualitative and semi-quantitative image analysis with flow cytometry) could provide an opportunity to gain further insights into the relative importance of microgliosis in pathological states and how it is affected by therapeutic interventions.

In conclusion, we have assessed alternative methods for the analysis of peripheral nerve injury-associated microgliosis in the spinal cord. We have defined optimal sensitivity and specificity of several immunohistochemical image analysis techniques and described a flow cytometric method that may be used alone or as a complementary technique for evaluation of spinal microgliosis.

## Figures and Tables

**Table 1:** Description of the qualitative scoring system for microglia activation state

Score	Activation state	Criteria
0	Resting (Fig. 1A)	Ramified cells with fine processes. Cells evenly distributed throughout the dorsal horn
1	Mild (Fig. 1B)	Ramified microglial, evenly spaced but with a slight increase in the number or density of cells
2	Moderate (Fig. 1C)	Microglia display shortened and clumpy processes, densely concentrated in the dorsal horn with slight overlap between individual microglia
3	Intense (Fig. 1D)	Microglia displaying hypertrophy of cell bodies and retraction of processes, with apparent amoeboid morphology, overlap between individual microglia and increased OX-42 immunoreactivity

**Table 2:** Description of parameters measured by standardised grid of detected microglial cells as visualised with OX42-IR

Measurement	Description
i Area %	Measure of percentage of total area covered by detected cells
ii Intercept H	Measure of either horizontal (H) or vertical (V) intersections on the grid by OX42-positive detected cell processes and nuclei (measured in terms of pixels). Indicative of possible changes in microglia morphology (dendritic vs. amoeboid)
iii Intercept V	
iv Count	Number of distinct detected features (individual and whole microglia cells) counted over all cellular areas falling within the grid
v Count/area	Combination of i and iv providing numerical and surface area density of immunoreactive cells within the grid

**Table 3:** Correlation between qualitative reference method and semi-quantitative image analysis paradigms

Method	CY3	DAB
GSI i	0.257	−0.535
GSI ii	0.091	−0.713*
GM i Area %	0.844*	0.859*
GM ii Intercept H	0.561	0.853*
GM iii Intercept V	0.564	0.862*
GM iv Count	0.603	0.614
GM v Count/area	0.452	−0.920*

Correlations based on comparison of microgliosis measured from ipsilateral L5 SNT spinal cord tissue in which microglial cells were identified by OX42-IR, using either fluorescent (CY3) or biotinylated (DAB) secondary visualisation. GSI, grey scale intensity measure (i, conventional grey scale; ii, expanded grey scale); GM, grid measurements. The Pearson correlation was used in correlating the semi-quantitative methods to the qualitative reference method. *Denotes significant correlations (*p* < 0.05).

**Table 4:** Specificity and sensitivity of immunohistochemical methods in detecting spinal microgliosis

Method	Sensitivity (%)		Specificity (%)	
	CY3	DAB	CY3	DAB
Categorical scale	100	100	63	100
Grey scale intensity (GSI) i Conventional	88	90	75	90
Grey scale intensity (GSI) ii Expanded	88	90	88	90
Grid measure (GM) i Area %	100	100	100	100
Grid measure (GM) ii Intercept H	88	100	100	100
Grid measure (GM) iii Intercept V	88	100	88	100
Grid measure (GM) iv Count	75	100	75	100
Grid measure (GM) v Count/area	75	100	75	100

The specificity and sensitivity of immunohistochemical methods in detecting microgliosis in ipsilateral spinal cord tissue from naïve vs. L5 SNT nerve-injured animals in which microglia was detected by labelling with OX42 and secondary visualisation including either fluorescence (Cy3) or biotinylated (DAB). Data are presented as percentages and are based on analysis of at least eight immunohistochemically stained spinal cord sections from each condition.
